# Supplementary material for: Adjuvant chemoradiotherapy versus chemotherapy or radiotherapy in advanced endometrial cancer: a systematic review and meta-analysis
Source: PeerJ. 2022 Nov 22;10:e14420. doi: 10.7717/peerj.14420 (PMC9695495; doi:10.7717/peerj.14420)
Supplement: Supplemental Information 2 [file peerj-10-14420-s002.docx]

| **Database** | **Search Terms** | **Hits** |
| --- | --- | --- |
| **Ovid EMBASE** | 1 endometrium cancer/  2 advance stage.mp.  3 stage III.mp.  4 stage IV.mp.  5 1 and 2  6 1 and 3  7 1 and 4  8 5 or 6 or 7  9 failure free survival/ or cancer specific survival/ or disease free survival/ or distant metastasis free survival/ or cancer free survival/ or local recurrence free survival/ or progression free survival/ or disease specific survival/ or metastasis free survival/ or local failure free survival/ or survival/ or local disease free survival/ or overall survival/ or recurrence free survival/ or distant recurrence free survival/  10 cancer chemotherapy/  11 cancer radiotherapy/ or radiotherapy/  12 radiation.mp. or radiation/  13 chemoradiation.mp. or chemoradiotherapy/  14 10 or 11 or 12 or 13  15 8 and 9 and 14  16 15 and "Article".sa_pubt. | 77 |
| **PUBMED** | ((((endometrial cancer[Abstract] AND advanced stage[Abstract]) OR (endometrial stage[Abstract] AND stage III[Abstract])) OR (endometrial cancer[Abstract] AND stage IV[Abstract])) AND (((survival[Abstract] OR overall survival[Abstract]) OR progression free survival[Abstract]) OR recurrence[Abstract])) AND (((chemotherapy[Abstract] OR chemoradiation[Abstract]) OR radiation[Abstract]) OR radiotherapy[Abstract]) | 26 |
| **SCOPUS** | ( TITLE-ABS-KEY ( advanced  AND  stage )  OR  TITLE-ABS-KEY ( stage  AND iii )  OR  TITLE-ABS-KEY ( stage  AND iv )  OR  TITLE-ABS-KEY ( stage  3 )  OR  TITLE-ABS-KEY ( stage  4 ) )  AND  ( TITLE-ABS-KEY ( endometrial  AND  cancer )  OR  TITLE-ABS-KEY ( endometrial  AND  neoplasm )  OR  TITLE-ABS-KEY ( endometrium  AND  carcinoma ) )  AND  ( TITLE-ABS-KEY ( postoperative )  OR  TITLE-ABS-KEY ( post-surgery ) )  AND  ( TITLE-ABS-KEY ( adjuvant  AND  therapy )  AND  TITLE-ABS-KEY ( chemoradiotherapy ) )  AND  ( TITLE-ABS-KEY ( radiotherapy )  OR  TITLE-ABS-KEY ( chemoradiotherapy ) )  AND  ( TITLE-ABS-KEY ( outcome )  OR  TITLE-ABS-KEY ( overall  AND  survival )  OR  TITLE-ABS-KEY ( recurrence )  OR  TITLE-ABS-KEY ( mortality )  OR  TITLE-ABS-KEY ( progression ) ) | 30 |
| **Ebscohost MEDLINE and CINAHL** | TX ( stage iii endometrial cancer or stage iv endometrial cancer or advanced endometrial cancer ) AND TX ( chemoradiotherapy or chemoradiation) AND TX ( chemotherapy or chemo or cancer treatment or radiotherapy or radiation therapy or therapeutic radiography ) AND TX ( survival or outcome or mortality or progression or recurrence ) | 92 |
| **The Cochrane Library** | TX ( stage iii endometrial cancer or stage iv endometrial cancer or advanced endometrial cancer ) AND TX ( chemoradiotherapy or chemoradiation) AND TX ( chemotherapy or chemo or cancer treatment or radiotherapy or radiation therapy or therapeutic radiography ) AND TX ( survival or outcome or mortality or progression or recurrence ) | 18 |
| **Web of Science** | 1. AB=endometrial cancer AND AB=advanced stage 2. AB=(endometrial cancer AND stage III) 3. AB=(endometrial cancer AND stage IV) 4. AB=(chemotheraphy OR chemoradiation OR radiation OR radiotherapy) 5. AB=(survival OR overall survival OR progression free survival) 6. #1 OR #2 OR #3 7. #6 AND #4 AND #5 8. #6 AND #4 AND #5 9. AB=(trial OR restrospective) 10. #9 AND #8 11. #9 AND #8 | 40 |
| **Total** |  | **283** |
