# Supplementary material for: Adjuvant chemoradiotherapy versus chemotherapy or radiotherapy in advanced endometrial cancer: a systematic review and meta-analysis
Source: PeerJ. 2022 Nov 22;10:e14420. doi: 10.7717/peerj.14420 (PMC9695495; doi:10.7717/peerj.14420)
Supplement: Supplemental Information 3 [file peerj-10-14420-s003.docx]

| **Author** | **Groups** | **Sample (N)** | **Treatment** | **Age (years)**  **Mean ± Standard deviation / Median (Interquartile range)** | **p** | **Stage IV**  **N (%)** | **p** | **Non endometrioid**  **N (%)** | **p** | **Grade 3**  **N (%)** | **p** | **5-year Overall survival (%)** | **5 years**  **Disease free survival (%)** | **Hazard Ratio**  **(95% Confidence interval)** | **p** |
| --- | --- | --- | --- | --- | --- | --- | --- | --- | --- | --- | --- | --- | --- | --- | --- |
| De Boer [18] | chemoradiotherapy | 152 | cisplatin 2 cycles and external beam radiotherapy and carboplatin + paclitaxel 4 cycles | not available | not available | 0(0) | - | not available | not available | not available | not available | 78.5 | - | overall survival: 0.63 (0.41–0.99) | overall survival: 0.043 |
|  | radiotherapy | 143 | external beam radiotherapy 48.6 gy | not available |  | 0(0) |  | not available |  | not available |  | 68.5 | - |  |  |
| Pichatechaiyoot [20] | chemoradiotherapy | 11 | cisplatin + adriamycin (64%) in chemotherapy-radiotherapy regimen (55%) radiotherapy-chemotherapy regimen (18%) | 56.1±12 | 0.934 | 0(0) | - | 1(9) | 0.028 | 6(55) | 0.455 | 75.0 | 38.9 | overall survival: 0.86 (0.18 - 4)  disease-free survival: 0.66 (0.18 -2.4) | overall survival: 0.85  disease-free survival: 0.74 |
|  | radiotherapy | 33 | vaginal brachytherapy or whole-pelvis radiotherapy, vaginal brachytherapy+whole-pelvis radiotherapy, | 57.2±10. |  | 0(0) |  | 1(3) |  | 21(64 |  | 71.6 | 52.5 |  |  |
| Albeesh [24] | chemoradiotherapy | 37 | carboplatin + paclitaxel 4-6 cycles and external beam radiotherapy | 64 (31-83) | 0.84 | 0(0) | - | 19 (54) | 0.01 | 25(37) | 0.006 | 61.0 | 51.0 | overall survival: 0.53 (0.28–1.00)  disease-free survival: 0.90 (0.45–1.79) | overall survival: 0.05  disease-free survival: 0.77 |
|  | radiotherapy | 67 | external beam radiotherapy 45 gy in 1.8 gy per fraction | 68 (42-89) |  | 0(0) |  | 17 (24) |  | 24(65) |  | 67.0 | 67.0 |  |  |
| Weldeen [23] | chemoradiotherapy | 175 | unspecified chemotherapy regimens and external beam radiotherapy | 62.9± 9.5 | not available | 0(0) | - | 85 (49) | not available | 109(62) | not available | 61.0 | - | overall survival: 0.58 (0.44-0.77) | overall survival: <0.001 |
|  | radiotherapy | 650 | external beam radiotherapy | 66.5±10.5 |  | 0(0) |  | 114 (18) |  | 262(40) |  | 55.0 | - |  |  |
| Lee [9] | chemoradiotherapy | 44 | caboplatin + paclitaxel or paclitaxel + doxorubicin + cyclophosphamide and external beam radiotherapy | 57.5 | 0.04 | 0(0) | - | 0 (0) | - | 19(43) | 0.14 | 90.0 | 79.0 | overall survival: 0.20 (0.05–0.75)  disease-free survival: 0.12 (0.03–0.49) | overall survival: 0.02  disease-free survival: <0.01 |
|  | radiotherapy | 18 | external beam radiotherapy 45 gy | 63 |  | 0(0) |  | 0 (0) |  | 3(17) |  | 67.0 | 63.0 |  |  |
| Secord (2013) [11] | chemoradiotherapy | 161 | paclitaxel + carboplatin or doxorubicin + cisplatin and whole-pelvis radiotherapy, ± extended filed ± vaginal brachytherapy in sandwich (38%) or chemotherapy-radiotherapy (43%) regimen | 60±10 | 0.005 | 0(0) | - | 59(47) | 0.66 | 84(52) | 0.2 | 90.0 | - | overall survival: 0.9 (0.28-3.33) | overall survival: 0.91 |
|  | radiotherapy | 45 | whole-pelvis radiotherapy, ± extended filed ± vaginal brachytherapy | 67±12 |  | 0(0) |  | 25(56) |  | 27(63 |  | 95.0 | - |  |  |
| Secord (2007) [21] | chemoradiotherapy | 83 | platinum + paclitaxel + adriamycin (85%) and whole-pelvis radiotherapy, + extended field ± vaginal brachytherapy | 63±9.6 | 0.11 | 24 (29) | <0.001 | 49(59) | 0.006 | 62(75) | 0.001 | 79.0 | 62.0 | overall survival:0.49 (0.28-0.85)  disease-free survival: 0.62 (0.39–0.97) | overall survival: 0.012  disease-free survival: 0.037 |
|  | radiotherapy | 171 | Whole abdomen irradiation (39%) or whole-pelvis radiotherapy, (35%) or whole-pelvis radiotherapy, and extended field ± vaginal brachytherapy (12%) | 65±10.8 |  | 21 (12) |  | 92(54) |  | 85(50) |  | 70.0 | 59.0 |  |  |
| Lim [25] | chemoradiotherapy | 41 | carboplatin + paclitaxel 6 cycles (61%) and external beam radiotherapy and vaginal brachytherapy | 51 (34-66) | 0.302 | 0 (0) | - | 0(0) | - | 14(34) | 0.006 | 89.3 | 87.2 | overall survival: 0.74(1.18-2.90)  disease-free survival: 0.87 (0.26-2.94) | overall survival: 0.666  disease-free survival: 0.822 |
|  | radiotherapy | 55 | external beam radiotherapy 45-50.4 gy and vaginal brachytherapy 10gy | 55 (30-85) |  | 0 (0) |  | 0(0) |  | 6(11) |  | 77.2 | 75.9 |  |  |
| Tai [22] | chemoradiotherapy | 28 | one of two types of chemotherapy and radiotherapy in sandwich or sequential regimen | not available | not available | not available | not available | 0(0) | - | 12(43) | not available | not available | - | overall survival: 0.78(0.25-2.37) | overall survival: 0.662 |
|  | radiotherapy | 87 | external beam radiotherapy 5040cgy ± vaginal brachytherapy | not available |  | not available |  | 0(0) |  | 24(28) |  | not available | - |  |  |
| Nakayama [28] | chemoradiotherapy | 26 | chemotherapy followed by radiotherapy (77%), radiotherapy followed by radiotherapy (23%) | 54±9.3 | not available | 0 | 0.016 | 24 | not available | 6 | not available | 77% | not available | overall survival: 0.21 (0.09-0.45) | overall survival: 0.0521 |
|  | radiotherapy | 20 | whole-pelvis radiotherapy, (50%), whole-pelvis radiotherapy, + para-aortic radiotherapy (46%) | 56±11.8 |  | 1 |  | 18 |  | 4 |  | 0% |  |  |  |
| Boothe [29] | chemoradiotherapy | 9595 | any sequential/concurrent chemotherapy and radiotherapy regimen | not available | not available | 0 (0) | - | 0 (0) | - | not available | not available | not available | - | overall survival: 0.67 (0.64-0.71) | overall survival: <0.01 |
|  | radiotherapy | 4486 | external beam radiotherapy, brachytherapy, or both | not available |  | 0 (0) |  | 0 (0) |  | not available |  | not available |  |  |  |
| Goodman [30] | chemoradiotherapy | 2070 | radiotherapy before chemotherapy or radiotherapy after chemotherapy | not available | not available | not available | not available | 0 (0) | - | 0 (0) | - | rt before ct: 73.3  rt after ct: 80.1 | - | overall survival: 0.78 (0.54-1.13) | overall survival: 0.18 |
|  | radiotherapy | 1267 | not stated | not available |  | not available |  | 0 (0) |  | 0 (0) |  | 64.5 |  |  |  |
| Kahramanoglu [26] | chemoradiotherapy | 544 | mostly carboplatin + paclitaxel + external beam radiotherapy ± vaginal brachytherapy | not available | not available | 0(0) | - | 0(0) | - | not available | not available | 72.8 | 62.0 | overall survival: 1.1 (0.51-2.38)  disease-free survival: 1.02 (0.76-1.39) | overall survival:0.80  disease-free survival:0.895 |
|  | radiotherapy | 179 | external beam radiotherapy ± vaginal brachytherapy | not available |  | 0(0) |  | 0(0) |  | not available |  | 77.1 | 67.0 |  |  |
| Wong [27] | chemoradiotherapy | 2522 | unspecified chemotherapy regimens + external beam radiotherapy or vaginal brachytherapy | 60 | <0.001 | 0(0) | - | not available | not available | 990 (37.6) | <0.001 | 72.6 | - | overall survival: 0.77 (0.67-0.89) | overall survival:0.0002 |
|  | radiotherapy | 1265 | external beam radiotherapy alone or vaginal brachytherapy or external beam radiotherapy ± vaginal brachytherapy | 64 |  | 0(0) |  | NA |  | 416 (15.8) |  | 63.9 | - |  |  |
