# Supplementary material for: Adjuvant chemoradiotherapy versus chemotherapy or radiotherapy in advanced endometrial cancer: a systematic review and meta-analysis
Source: PeerJ. 2022 Nov 22;10:e14420. doi: 10.7717/peerj.14420 (PMC9695495; doi:10.7717/peerj.14420)
Supplement: Supplemental Information 4 [file peerj-10-14420-s004.docx]

| **Author** | **Groups** | **Sample (n)** | **Treatment** | **Age (years)**  **Mean ± standard deviation / median (interquartile range)** | **P** | **Stage iv**  **N (%)** | **P** | **Non endo-metrioid**  **N (%)** | **P** | **Grade 3**  **N (%)** | **P** | **5-year overall survival (%)** | **5 years**  **disease-free survival (%)** | **Hazard ratio**  **(95% confidence interval)** | **P** |
| --- | --- | --- | --- | --- | --- | --- | --- | --- | --- | --- | --- | --- | --- | --- | --- |
| Matei [19] | chemoradiotherapy | 294 | cisplatin + external beam radiotherapy + cisplatin | not available | not available | 3(0.01) | not available | not available | not available | not available | not available | - | not available | disease-free survival: 1.02 (0.67-1.57) | disease-free survival: 0.92 |
|  | chemotherapy | 275 | cisplatin + paclitaxel | not available |  | 3(0.01) |  | not available |  | not available |  | - | not available |  |  |
| Pichatechaiyoot [20] | chemoradiotherapy | 11 | cisplatin + adriamycin (64%) in chemotherapy-radiotherapy regimen (55%) radiotherapy-chemotherapy regimen (18%) | 56.1**±**12.0 | 0.93 | 0(0) | - | 1 (9) | 0.028 | 6 (55) | 0.455 | 75.0 | 38.9 | overall survival: 0.79 (0.11-5.55)  disease-free survival: 0.94 (0.21-4.35) | overall survival: 0.81  disease-free survival: 0.97 |
|  | chemotherapy | 10 | cisplatin + adriamycin (50%) or carboplatin + paclitaxel (30%) or cisplatin + cyclophoshamide (20%) | 54.3**±**9.5 |  | 0(0) |  | 3 (30) |  | 6 (60) |  | 60.0 | 57.1 |  |  |
| Weldeen [23] | chemoradiotherapy | 175 | unspecified chemotherapy regimens and external beam radiotherapy | 62.9± 9.5 | not available | 0(0) | - | 85(49) | not available | 109(62 | not available | 61.0 | - | overall survival: 0.67 (0.49-0.92) | overall survival: 0.014 |
|  | chemotherapy | 158 | unspecified chemotherapy regimens | 66.5± 10.5 |  | 0(0) |  | 128(81) |  | 114(72 |  | 39.0 | - |  |  |
| Lester-coll [10] | chemoradiotherapy | 3479 | unspecified regimens | 61(55-68) | <0.001 | 23 (2) | <0.001 | 2884 (70) | <0.001 | 1953 (56) | <0.001 | 70.0 | - | overall survival: 0.63 (0.57-0.69) | overall survival: <0.001 |
|  | chemotherapy | 6358 | unspecified regimens | 62(56-70) |  | 363 (11) |  | 5332 (76) |  | 4270 (67) |  | 55.0 | - |  |  |
| Secord  (2013) [11] | chemoradiotherapy | 161 | paclitaxel + carboplatin or doxorubicin + cisplatin and whole-pelvis radiotherapy ± extended filed ± vaginal brachytherapy in sandwich (38%) or chemotherapy-radiotherapy (43%) regimen | 60**±**10 | 0.005 | 0 (0) | - | 59 (36) | 0.66 | 84 (52) | 0.009 | 90.0 | - | overall survival: 0.25 (0.1-0.625) | overall survival: 0.004 |
|  | chemotherapy | 46 | carboplatin + paclitaxel or doxorubicin + paclitaxel or other regimens | 60**±**11 |  | 0 (0) |  | 14 (30) |  | 12 (27) |  | 78.0 | - |  |  |
| Secord (2007) [21] | chemoradiotherapy | 83 | platinum + paclitaxel + adriamycin (85%) and whole-pelvis radiotherapy + extended field ± vaginal brachytherapy | 63 (9.6) | 0.007 | 24(29) | <0.001 | 49(59) | 0.011 | 62(75) | 0.216 | 79.0 | 62.0 | overall survival; 0.62 (0.34-1.14)  disease-free survival: 0.64 (0.39-1.04) | overall survival: 0.122  disease-free survival: 0.076 |
|  | chemotherapy | 102 | platinum + adriamycin + cyclophosphamide (33%) or platinum + paclitaxel + adriamycin (58%) | 67 (9.7) |  | 75(74) |  | 81(79) |  | 85(84) |  | 33.0 | 19.0 |  |  |
| Tai [22] | chemoradiotherapy | 28 | one of two types of chemotherapy and radiotherapy in sandwich or sequential regimen | not available | not available | not available | not available | not available | not available | 12(43) | not available | not available | not available | overall survival: 0.49 (0.16-1.50) | overall survival: 0.191 |
|  | chemotherapy | 79 | platinum + paclitaxel, anthracycline, cyclophosphamide, or ifosdamide | not available |  | not available |  | not available |  | 31(39) |  | not available | not available |  |  |
| Nakayama [28] | chemoradiotherapy | 26 | chemotherapy followed by radiotherapy (77%), radiotherapy followed by radiotherapy (23%) | 54±9.3 | not available | 0 | 0.016 | 24 | not available | 6 | not available | 77% | not available | overall survival: 0.78 (0.43-1.41) | overall survival: 0.0345 |
|  | chemotherapy | 30 | cisplatin + adriamycin + cyclophospha-  mide (73%), paclitaxel + carboplatin (13%) | 53±9.7 |  | 7 |  | 28 |  | 8 |  | 60% |  |  |  |
| Boothe [29] | chemoradiotherapy | 9595 | any sequential/concurrent chemotherapy and radiotherapy regimen | not available | not available | 0 (0) | - | 0 (0) | - | not available | not available | not available | - | overall survival: 0.69 (0.66-0.72) | overall survival: <0.01 |
|  | chemotherapy | 6946 | not stated | not available |  | 0 (0) |  | 0 (0) |  | not available |  | not available |  |  |  |
| Goodman [30] | chemoradiotherapy | 2070 | radiotherapy before chemotherapy or radiotherapy after chemotherapy | not available | not available | not available | not available | 0 (0) | - | 0 (0) | - | radiotherapy before chemotherapy: 73.3  radiotherapy after chemotherapy: 80.1 | <0.001 | overall survival: 0.89 (0.63-1.26) | overall survival: 0.5087 |
|  | chemotherapy | 2465 | not stated | not available |  | not available |  | 0 (0) |  | 0 (0) |  | 68.9 |  |  |  |
| Kahramanoglu [26] | chemoradiotherapy | 544 | mostly carboplatin + paclitaxel + external beam radiotherapy ± vaginal brachytherapy | not available | not available | 0(0) | - | 0(0) | - | not available | not available | 72.8 | 62.0 | overall survival: 0.87 (0.65-1.18)  disease-free survival: 0.75 (0.57-0.99) | overall survival:0.34  disease-free survival:0.039 |
|  | chemotherapy | 242 | mostly carboplatin + paclitaxel | not available |  | 0(0) |  | 0(0) |  | not available |  | 69.8 | 53.7 |  |  |
| Wong [27] | chemoradiotherapy | 2522 | unspecified chemotherapy regimens + external beam radiotherapy or vaginal brachytherapy | 60 | <0.001 | 0(0) | - | not available | not available | 990(37.6) | <0.001 | 72.6 | - | overall survival: 0.62 (0.55-0.71) | overall survival:0.000 |
|  | chemotherapy | 1533 | unspecified chemotherapy regimens | 61 |  | 0(0) |  | not available |  | 678(25.7) |  | 64.4 | - |  |  |
| Xiang [31] | chemoradiotherapy | 5311 | unspecified chemotherapy regimens + external beam radiotherapy | not available | not available | not available | not available | 1765 (33) | not available | not available | not available | 70 | - | overall survival: 0.83 (0.77-0.89) | overall survival:0.000 |
|  | chemotherapy | 7959 | unspecified chemotherapy regimens | not available |  | not available |  | 3282 (41) |  | not available |  | 62 | - |  |  |
