# Supplementary material for: Adjuvant chemoradiotherapy versus chemotherapy or radiotherapy in advanced endometrial cancer: a systematic review and meta-analysis
Source: PeerJ. 2022 Nov 22;10:e14420. doi: 10.7717/peerj.14420 (PMC9695495; doi:10.7717/peerj.14420)
Supplement: Supplemental Information 5 [file peerj-10-14420-s005.docx]

|  | **Random sequence generation (Selection bias)** | **Allocation concealment (Selection bias)** | **Blinding of participants and personnel (performance bias)** | **Blinding of researchers conducting outcome assessments (detection bias)** | **Incomplete outcome data (attrition bias)** | **Selective reporting (reporting bias)** | **Other sources of bias** |
| --- | --- | --- | --- | --- | --- | --- | --- |
| PORTEC-3 [18] | 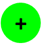 | 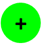 | 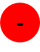 | 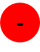 | 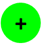 | 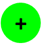 | 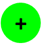 |
| GOG 258 [19] | 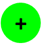 | 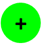 | 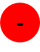 | 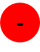 | 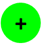 | 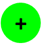 | 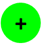 |
