# Supplementary material for: Adjuvant chemoradiotherapy versus chemotherapy or radiotherapy in advanced endometrial cancer: a systematic review and meta-analysis
Source: PeerJ. 2022 Nov 22;10:e14420. doi: 10.7717/peerj.14420 (PMC9695495; doi:10.7717/peerj.14420)
Supplement: Supplemental Information 6 [file peerj-10-14420-s006.docx]

| **No** | **First Author** | **Year of Publication** | **Study Location** | **Study Period** | **Study Design** | **Selection** | **Comparability** | **Outcome** | **Total NOS Scale** |
| --- | --- | --- | --- | --- | --- | --- | --- | --- | --- |
| 1 | Secord [21] | 2007 | United States | 1975 - 2006 | Retrospective Observational | ******* | ****** | ***** | 6 |
| 2 | Lim [25] | 2020 | Singapore | Januari 2000 – December 2010 | Retrospective Observational | ****** | ****** | ****** | 6 |
| 3 | Pichatechaiyoot [20] | 2014 | Thailand | January 2003 – December 2012 | Retrospective Observational | ****** | ****** | ***** | 5 |
| 4 | Albeesh [24] | 2018 | Canada | 1990 - 2012 | Retrospective Observational | ****** | ****** | ****** | 6 |
| 5 | Weldeen [23] | 2020 | Netherland | January 1, 2005 – December 31, 2016 | Retrospective Observational | ******* | ****** | ****** | 7 |
| 6 | Lee [9] | 2012 | United States | January 1, 1995 – December 31, 2009 | Retrospective Observational | ******* | ****** | ****** | 7 |
| 7 | Lester-Coll [10] | 2016 | United States | 2004 - 2012 | Retrospective Observational | ******** | ****** | ****** | 8 |
| 8 | Secord [11] | 2013 | United States | 1975 - 2006 | Retrospective Observational | ******* | ****** | ***** | 6 |
| 9 | Tai [22] | 2019 | Taiwan | January 1, 2000 – August 31, 2017 | Retrospective Observational | ******* | ****** | ****** | 7 |
| 10 | Nakayama [28] | 2010 | Japan | 1985 - 2007 | Retrospective Observational | ******* |  | ****** | 5 |
| 11 | Goodman [30] | 2019 | United States | 2004 - 2015 | Retrospective Observational | ******* | ****** | ****** | 7 |
| 12 | Boothe [29] | 2016 | United States | 2004 - 2013 | Retrospective Observational | ******* | ****** | ****** | 7 |
| 13 | Kahramanoglu [26] | 2019 | Kazakhstan | January 1, 1998 - August 31, 2018 | Retrospective Observational | ******** | ****** | ****** | 8 |
| 14 | Xiang [31] | 2019 | United States | 2004 - 2016 | Retrospective Observational | ******* | ****** | ****** | 7 |
| 15 | Wong [27] | 2016 | United States | 2004 - 2011 | Retrospective Observational | ******** | ****** | ****** | 8 |
