# Supplementary figures and images for: Adjuvant chemoradiotherapy versus chemotherapy or radiotherapy in advanced endometrial cancer: a systematic review and meta-analysis
Source: PeerJ. 2022 Nov 22;10:e14420. doi: 10.7717/peerj.14420 (PMC9695495; doi:10.7717/peerj.14420)

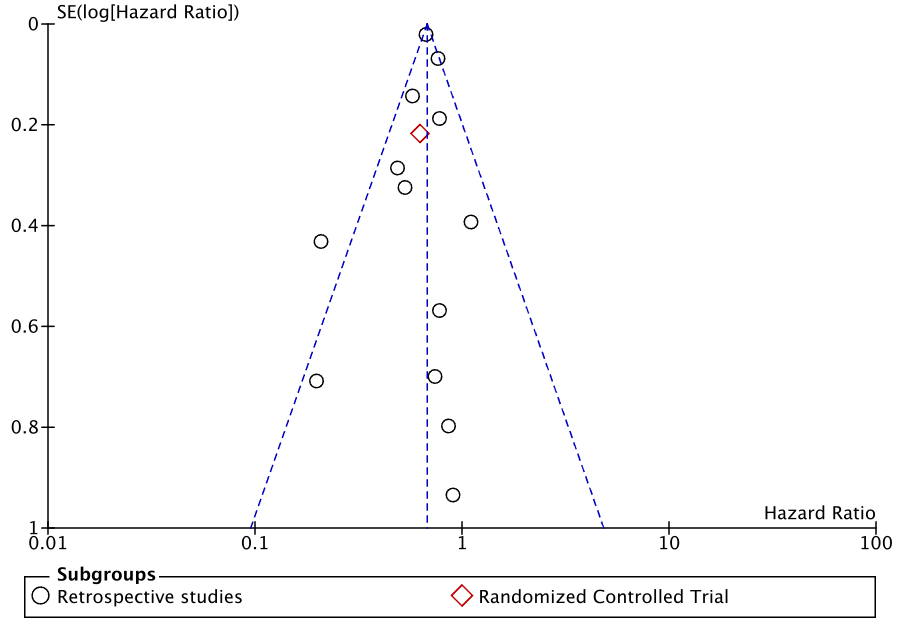

Supplement: Supplemental Information 7 [file peerj-10-14420-s007.png]

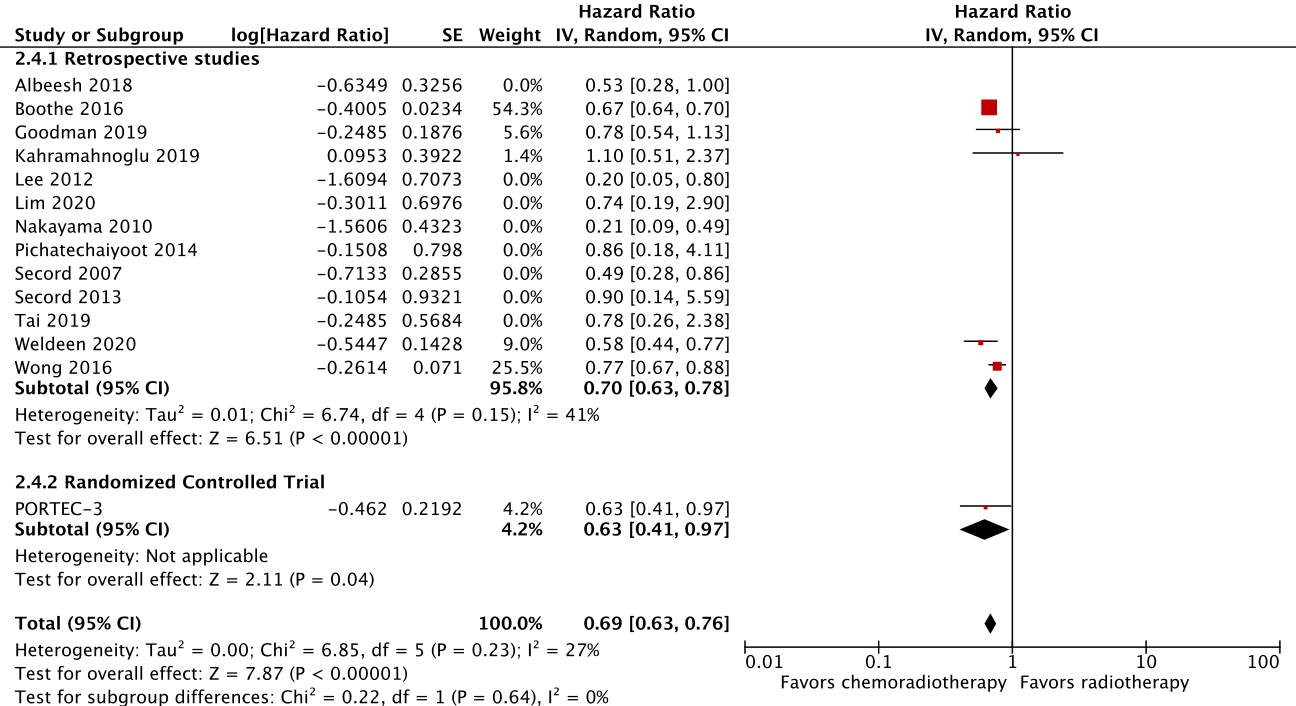

Supplement: Supplemental Information 8 [file peerj-10-14420-s008.png]

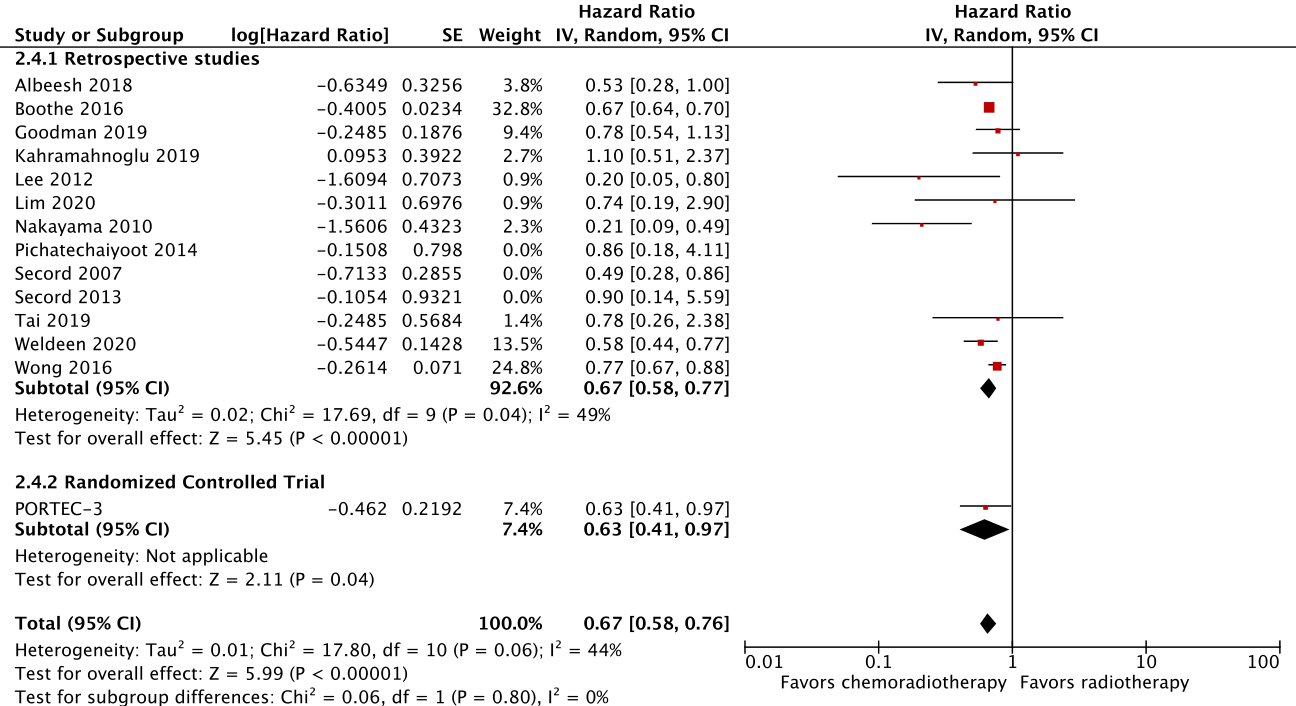

Supplement: Supplemental Information 9 [file peerj-10-14420-s009.png]

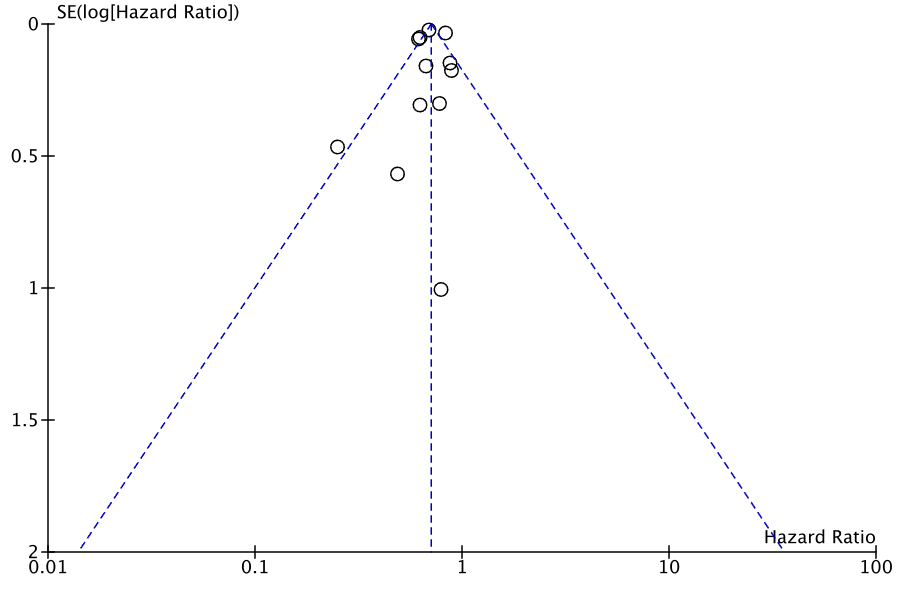

Supplement: Supplemental Information 10 [file peerj-10-14420-s010.png]

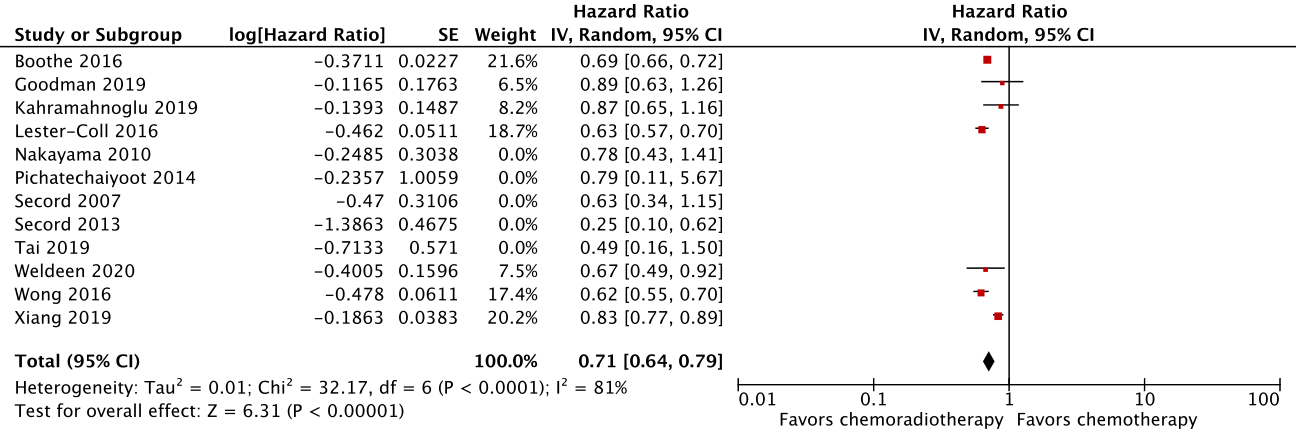

Supplement: Supplemental Information 11 [file peerj-10-14420-s011.png]

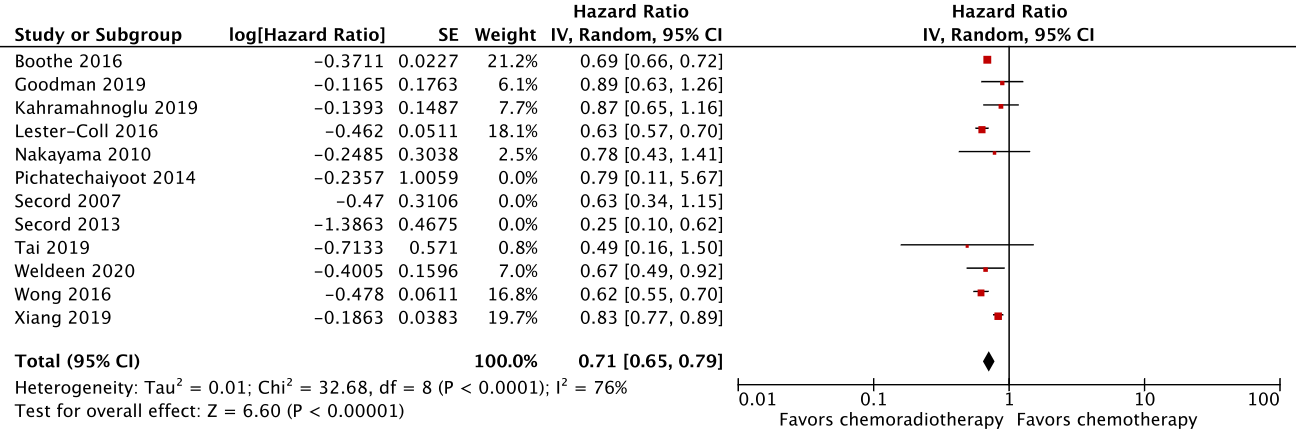

Supplement: Supplemental Information 12 [file peerj-10-14420-s012.png]
