# Supplementary material for: Adjuvant chemoradiotherapy versus chemotherapy or radiotherapy in advanced endometrial cancer: a systematic review and meta-analysis
Source: PeerJ. 2022 Nov 22;10:e14420. doi: 10.7717/peerj.14420 (PMC9695495; doi:10.7717/peerj.14420)
Supplement: Supplemental Information 13 [file peerj-10-14420-s013.docx]

**The rationale for conducting the systematic review / meta-analysis**

**Answer:** Radiotherapy has been used as adjuvant therapy for patients with advanced-stage endometrial cancer for its ability to control local recurrence. However, radiotherapy demonstrated limited impact on distal recurrence. Thus, physicians have considered the role of adjuvant chemotherapy. Studies showed that chemotherapy alone also have a limited impact on preventing pelvic recurrence, which accounts for only 18% as the first relapse site in the advanced stage. To overcome the pitfall of monotherapy, clinicians have started implementing combination adjuvant therapy. The clinical effectiveness of chemoradiotherapy in advanced-stage endometrial cancer however, has not yet been established. We conducted a systematic review and meta-analysis to determine the clinical effectiveness of chemoradiotherapy as compared to radiotherapy or chemotherapy only as adjuvant treatment for women with advanced-stage endometrial cancer.

**The contribution that it makes to knowledge in light of previously published related reports, including other meta-analyses and systematic reviews**

**Answer:** To our knowledge, this is the first systematic review and meta-analysis evaluating the clinical effectiveness of adjuvant chemoradiotherapy as compared to radiotherapy or chemotherapy only in advanced-stage endometrial carcinoma. The result of this study will help clinician in determining the best option for adjuvant therapy. Our study showed that chemoradiotherapy can significantly improve overall survival compared to chemotherapy or radiotherapy alone. Hence, adjuvant chemoradiotherapy becomes a reasonable treatment option in advanced-stage endometrial cancer.
